# Knowledge of acute respiratory infection in under-fives and homebased practices by their caregivers in an urban community in southern Nigeria

**DOI:** 10.7196/AJTCCM.2018.v24i4.188

**Published:** 2018-12-20

**Authors:** W O Osarogiagbon, A R Isara

**Affiliations:** 1 Department of Child Health, School of Medicine, University of Benin, Benin City, Nigeria; 2 Department of Community Health, School of Medicine, University of Benin, Benin City, Nigeria

**Keywords:** knowledge, under-five caregivers, home-based practice

## Abstract

**Background:**

Acute respiratory infection (ARI) is a common respiratory problem in Nigeria and spans a spectrum of infections, from the common cold to pneumonia. ARI is the leading cause of mortality in children under 5 years of age, with the majority of deaths occurring in developing countries.

**Objectives:**

To assess the knowledge of and the home-based practices used by caregivers of under-fives during episodes of ARI.

**Methods:**

A descriptive cross-sectional study was conducted among an urban community in Edo State, Nigeria. Participants were selected using a multistage sampling technique. A pretested researcher-administered questionnaire was used for data collection.

**Results:**

Of the 346 participating caregivers, the majority had poor knowledge of ARI-related aspects and only some respondents showed good or fair knowledge. Specifically, only 12 respondents (3.5%) showed good knowledge of ARI symptoms and only nine (2.6%) showed good knowledge of the danger signs associated with ARIs. The majority of respondents (n=163; 81.1%) used shea butter oil (orioyo) as a home remedy.

**Conclusion:**

Despite a high level of awareness of ARI among caregivers in the sampled community, a poor level of knowledge of the symptoms, causes and danger signs of ARI was observed. Various homemade preparations and remedies were used to treat ARIs.

## Background

Acute respiratory infection (ARI) has been a leading cause of underfive mortality in the last decade and has been responsible for about a fifth of the total number of deaths in this age group worldwide.^[1,2]^ Cases in developing countries, where up to 28% of the total number of the under-five mortalities are due to ARI, contribute greatly to the global statistic.^[1,3-5]^ The high number of under-five mortalities due to ARI may be associated with factors such as overcrowding, poor housing, poor breastfeeding habits and nutrition and, in some cases, incomplete or absent immunisation.^[1,6-10]^ Apart from encouraging the spread of causative organisms, some of these conditions may also make patients prone to developing severe ARIs such as pneumonia, and so worsen the outcome of the infection.^[1,6-10]^

Most of the deaths associated with ARI are indeed due to pneumonia, frequently caused by bacteria (about 70% of cases) such as *Streptococcus pneumoniae, S. aureus, Haemophilus influenzae, H. influenzae type B* and *Klebsiella pneumoniae*. ^[2,10-12]^ Viruses and other agents are responsible for the remaining cases.

Under-fives with ARI commonly present with fever and a cough and may develop rhinorrhoea.^[13,14]^ The nasal discharge is initially free flowing and clear but may later become purulent, especially when there is a superimposed infection.^[13–16]^ Many under-five ARI patients develop breathing difficulties. Some may produce abnormal sounds (e.g. grunting), become cyanosed or even convulse.^[13,14]^ These symptoms are usually clear indicators of a respiratory infection and can be useful in early diagnosis.^[13–16]^

Several cultural and socioeconomic factors influence caregivers’ approach to home-based care of under-fives with ARI.^[17–22]^ In certain cultures, children with breathing difficulties undergo herbal scarification. Cough is also often considered an illness itself rather than a symptom of an underlying condition, and traditional remedies may be used as treatment. To improve the outcome of communitybased practice, family caregivers need to be able to recognise early symptoms of ARI and avoid the use of potentially harmful homemade or traditional remedies.^[15,16]^

The outcome of ARI among under-fives in Nigeria remains poor and studies show that the correct home treatment of ARI needs to be emphasised to ensure a better outcome during episodes.^[9,10,12,17,18,23]^ To our knowledge, the approach to handling an ARI episode has not been studied among caregivers of under-fives in Benin City, Nigeria. Our study evaluated local caregivers’ home-based care practices in relation to findings from other areas^[17,18,20-23]^ to establish the knowledge regarding ARI and preventive measures.

## Methods

The study was conducted in Uselu 2, one of the 10 wards in the Egor Local Government Area of Edo State, Nigeria. The area occupies 93 km² and the current population is estimated at close to 430 000.^[24]^

The study was a cross-sectional community-based survey among caregivers of under-fives. All participants gave their consent to participation. Women of childbearing age but who did not have children were excluded from the study. Caregivers of under-fives with chronic disease or congenital malformations were also excluded, as these conditions may increase the rate of ARI in this group and affect the caregiver’s experience compared with that of the general population.

A multistage sampling technique was employed. Uselu 2 was selected from the 10 wards of the Egor Local Government Area using a simple random ballot. In the next stage, the Edaiken community was selected randomly from the five communities in the ward. This was followed by the selection of houses or households, which involved enumeration of the Edaiken community based on the existing primary healthcare numbering system. This step yielded a total of 1 250 houses as the sample frame. With a minimum sample size of 341, the sampling ratio was set at 4 (1 250/341 = 3.7 ≈ 4) and therefore every fourth house was sampled. If more than one household with an under-five resided in a sampled house, simple balloting was used to select only one household. Similarly, if no under-five resided in a selected house, or if the caregiver did not agree to be interviewed or was not available, the next available house with an under-five was selected. In the case of households with more than one child under five, caregivers were asked to base their responses only on experiences with the youngest.

A pretested researcher-administered questionnaire was completed during an interview with the respondents. The questionnaire included a preamble and an introduction to explain the reason for the study and the respondents were assured of confidentiality. Three data sections followed to determine:

- the socioeconomic status of the respondents (Section A)
- the knowledge of caregivers regarding various aspects of ARI (Section B)
- traditional, religious or cultural practices followed by caregivers during an ARI episode (Section C).

### Data analysis

Data captured with the questionnaire were entered into a spreadsheet and exported to SPSS (version 20.0) for analysis. The entered data were checked for inconsistency and errors. Continuous variables were summarised and categorical variables were grouped. Frequencies and means were determined for sociodemographic characteristics of the respondents.

In a bivariate analysis, chi-square tests were used to determine the association between selected sociodemographic characteristics of the caregivers and their knowledge of: ARI and its causes; symptoms and signs of ARI; and danger signs associated with ARI. The knowledge of ARI was assessed according to questions in various subsections related to the causes, symptoms and danger signs of ARI. Sections regarding the causes of ARI and danger signs of ARI each had five questions, with one correct answer option for each. A score of ≥3 was considered to reflect good knowledge. A score of 2 was considered to reflect fair knowledge and a score of ≤1 was interpreted as showing poor knowledge. A *p*-value of ≤0.05 was considered statistically significant.

### Ethical considerations

Institutional approval for the study was given by the Ethics and Research Committee of the University of Benin Teaching Hospital. Community consent was obtained from the *Odionwere* (community head) and elders of the Edaiken community. All respondents gave their written consent to participate.

## Results

Of the 346 respondents in the study, 46 (13.3%) had good knowledge of the symptoms of ARI, 147 (42.5%) had fair knowledge and 153 (44.2%) had poor knowledge (Table 1). Only 12 respondents (3.5%) had good knowledge about the causes of ARI, with the rest (96.5%) all showing poor knowledge. With regard to knowledge about danger signs associated with ARI, 9 (2.6%) and 16 (4.6%) respondents showed good or fair knowledge, respectively, whereas 321 (92.8%) respondents had poor knowledge.

**Table 1:** Knowledge of symptoms, causes and danger signs of acute respiratory infection

**Knowledge level**	**Frequency (N=346), n(%)**
**Symptoms of ARI**	
Good	46 (13.3)
Fair	147 (42.5)
Poor	153 (44.2)
Causes of ARI*	
Good	12 (3.5)
Poor	334 (96.5)
Danger signs and symptoms of ARI*	
Good	9 (2.6)
Fair	16 (4.6)
Poor	321 (92.8)

ARI = acute respiratory infection

*Multiple responses.

### Knowledge of the symptoms of acute respiratory infection

The associations between sociodemographic variables and knowledge of ARI symptoms are described in Table 2. The association between age group and knowledge of ARI symptoms was statistically significant (p=0.001), with younger respondents showing better knowledge.

**Table 2:** Sociodemographic characteristics and caregivers’ knowledge of symptoms of acute respiratory infection

	**Knowledge level, n (%)**			
**Variables**	**Poor**	**Fair**	**Good**	***p*-value**
**Age (years)**				0.001
≤30 (N=193)	73 (37.8)	83 (43.0)	37 (19.2)	
>30 (N=153)	80 (52.3)	64 (41.8)	9 (5.9)	
**Gender**				0.042
Male (N=45)	13 (28.9)	22 (48.9)	10 (22.2)	
Female (N=301)	140 (46.5)	125 (41.5)	36 (12.0)	
**Educational status**				0.079
Primary (N=53)	22 (41.5)	22 (41.5)	9 (17.0)	
Secondary (N=209)	92 (44.0)	97 (46.4)	20 (9.6)	
Tertiary (N=84)	39 (46.4)	28 (33.3)	17 (20.2)	
**Socioeconomic status**				0.130
Upper (N=88)	35 (50.0)	36 (25.0)	17 (25.0)	
Middle (N=199)	95 (47.7)	86 (43.2)	18 (9.1)	
Lower (N=59)	23 (39.0)	25 (42.4)	11 (18.6)	

The relationship between the gender of the caregiver and knowledge level was statistically significant (p=0.042), with 28.9% of male caregivers showing poor knowledge compared with 46.5% of female caregivers in this knowledge category.

Relatively few respondents showed good knowledge of the symptoms of ARI, regardless of educational status: a similar proportion of respondents with primary education showed good knowledge (17.0%) compared with respondents with tertiary education (20.2%). Likewise, a similar proportion of respondents showed poor knowledge of the symptoms of ARI across all three categories of educational status. In the group with secondary education, a similar proportion of respondents showed fair or poor knowledge (46.4% and 44.0%, respectively). The association between educational status of caregivers and knowledge of symptoms was not statistically significant (p=0.079).

A relatively larger proportion of respondents with good knowledge of the symptoms of ARI had a high socioeconomic status (25.0%), whereas a similar proportion of respondents of a mid-level or low socioeconomic status had fair knowledge (43.2% and 42.4%, respectively). Unexpectedly, however, 50.0% of the respondents of a high socioeconomic status showed poor knowledge, which is similar to the proportion of respondents from the middle socioeconomic group. There was no statistically significant association between knowledge level and socioeconomic status (p=0.130).

### Knowledge of the causes of acute respiratory infection

The associations between sociodemographic variables and knowledge of the causes of ARI are shown in Table 3. The majority of respondents had poor knowledge of the causes of ARI, regardless of age (94.8% and 99.7% among respondents ≤30 years and >30 years, respectively). However, of the 12 respondents who showed good knowledge, a relatively larger proportion were younger than 30 years (n=10; 83.3%). The association between the age of caregivers and knowledge of the causes of ARI was not statistically significant (p=0.051).

**TABLE 3:** Sociodemographic characteristics and caregivers’ knowledge of the causes of acute respiratory infection

	**Knowledge level, n (%)**		
**Variables**	**Poor**	**Good**	***p*-value**
**Age (years)**			0.051
≤30 (N=193)	183 (94.8)	10 (5.2)	
>30 (N=153)	151 (99.7)	2 (0.3)	
**Gender**			0.209
Male (N=45)	42 (93.3)	3 (6.7)	
Female (N=301)	292 (97.0)	9 (3.0)	
**Educational status**			0.014
Primary (N=53)	51 (96.2)	2 (3.8)	
Secondary (N=209)	206 (98.6)	3 (1.4)	
Tertiary (N=84)	77 (91.7)	7 (8.3)	
**Socioeconomic status**			0.126
Upper (N=88)	82 (93.8)	6 (6.2)	
Middle (N=199)	196 (98.5)	3 (1.5)	
Lower (N=59)	56 (94.9)	3 (5.1)	

Less than 10% of respondents in each of the gender groups had good knowledge of the causes of ARI, although proportionally more male caregivers had good knowledge (n=3/45; 6.7%) than female caregivers (n=9/292; 3.0%). The association between gender and knowledge of the causes of ARI was not statistically significant (p=0.209).

The majority of respondents (n=209) had secondary education, while about a fifth (n=77) had tertiary education. Of the respondents with secondary education, only three (1.4%) had good knowledge. Seven of the respondents with tertiary education (8.3%) had good knowledge. A larger proportion of respondents with tertiary education had good knowledge than in the group with secondary education. The association between educational status of caregivers and knowledge of the causes of ARI was statistically significant (p=0.014). In addition, the majority of respondents had poor knowledge of the causes of ARI regardless of their socioeconomic status. However, this relationship was not statistically significant (p=0.126).

### Knowledge of the danger signs associated with acute respiratory infection

The majority of respondents had poor knowledge, regardless of age (96.4% and 87.8% for those ≤30 years and >30 years, respectively) (Table 4). Although only a small number of respondents showed fair or good knowledge, those older than 30 years constituted a relatively larger proportion (e.g. of the nine respondents with good knowledge, eight were older than 30 years). This association between age and knowledge of the danger signs of ARI was statistically significant (p=0.007).

**Table 4:** Sociodemographic characteristics and caregivers’ knowledge of the danger signs associated with severe acute respiratory infection

	**Knowledge level, n (%)**			
**Variables**	**Poor**	**Fair**	**Good**	***p*-value**
**Age (years)**				0.007
≤30 (N=193)	186 (96.4)	6 (3.1)	1 (0.5)	
>30 (N=153)	135 (87.8)	10 (6.8)	8 (6.4)	
**Gender**				0.133
Male (N=45)	45 (100.0)	0 (0.0)	0 (0.0)	
Female (N=301)	276 (91.7)	16 (5.3)	9 (3.0)	
**Educational status**				0.356
Primary (N=53)	52 (98.1)	1 (1.9)	0 (0.0)	
Secondary (N=209)	194 (92.8)	10 (4.8)	5 (2.4)	
Tertiary (N=84)	75 (89.3)	5 (6.0)	4 (4.8)	
**Socioeconomic status**				0.057
Upper (N=88)	77 (87.5)	6 (6.8)	5 (5.7)	
Middle (N=199)	186 (93.5)	9 (4.5)	4 (2.0)	
Lower (N=59)	58 (98.3)	1 (1.7)	0 (0.0)	

Although all the male caregivers had poor knowledge of this aspect, the association between the gender of caregivers and their knowledge of the danger signs of severe ARI was not statistically significant (p=0.133), mainly owing to the sheer difference in the number of male and female caregivers. Although few respondents overall showed good or fair knowledge of this aspect when their educational status was considered, the relative proportion of respondents with good (0.0%, 2.4% and 4.8%) or fair knowledge (1.9%, 4.8% and 6.0%) increased as educational status moved from primary to tertiary level. The opposite trend was seen among respondents with poor knowledge, as the relative proportions of respondents with poor knowledge decreased with increasing educational level. However, the relationship between educational status and knowledge level was not significant (p=0.356).

When grouped according to socioeconomic status, the majority of respondents were considered to have a mid-level socioeconomic status. There was no significant association between socioeconomic status and knowledge of the danger signs of severe ARI (p=0.057), although the majority of respondents with good knowledge had a higher socioeconomic status.

### Home-based practices

A large proportion of respondents (n=163; 81.1%) indicated that they use shea butter oil (*orioyo*). Respondents also indicated that they apply Mentholatum to the nose (n=83; 41.3%), use palm kernel oil (n=55; 27.4%) or so-called scent leaf (*Ocimum gratissimum*) (n=12; 6.0%), or apply kerosene to the nose (n=35; 17.4%). One respondent (0.5%) indicated the application of palm oil to the nose as a remedy.

When asked about the use of home-based procedures, 223 respondents (64.5%) indicated that they suck mucus orally from the nose, while 26 (7.5%) used cotton buds to clean mucus from the nose. Close to a third (n=102; 29.6%) indicated that they stop using a fan and 96 (27.7%) indicated dressing the child in several layers of clothes. Steam inhalation with Metholatum was used by 77 respondents (22.3%), whereas <1% of respondents (n=3) used steam inhalation with herbs. Less than 1% of respondents (n=2) indicated the use of other methods (Table 5).

**Table 5:** Home-based practices in treating acute respiratory infections

**Practice**	**Frequency (N=346), *n*(%)**
Use of traditional remedies (N=192)*	
Apply shea butter oil (orioyo)	163 (81.1)
Apply Mentholathum	83 (41.3)
Apply palm kernel oil	55 (27.4)
Apply kerosene to nose	35 (17.4)
Apply scent leaf (Ocimum gratissimum)	12 (6.0)
Apply palm oil to nose	1 (0.5)
Native procedures performed at home*	
Suck mucus orally from nose	223 (64.5)
Stop using the fan	102 (29.6)
Dressing the child in several layers of clothes	96 (27.7)
Steam inhalation with Mentholatum	77 (22.3)
Clean mucus from nose using cotton buds	26 (7.5)
Steam inhalation with herbs	3 (0.9)
Other^†^	2 (0.6)

*Multiple responses

^†^These included the use of a handkerchief and application of a rub or shea butter oil to the nose.

## Discussion

A fairly low level of knowledge regarding the symptoms, causes and danger signs of ARI was seen among the caregivers in this study. Only a few respondents knew that ARI could be caused by a viral infection, which could result in many not being aware of the possible complications that may arise from apparently simple cases of ARI. ARI can lead to life-threatening septicaemia or meningitis.

The low level of knowledge of the aspects investigated in this study could be due to a lack of proper health education by healthcare providers. This may be detrimental to appropriate home treatment of ARI. Poor knowledge, specifically of symptoms and danger signs of ARI, may result in caregivers failing to visit health facilities timely. This may be detrimental to home management and outcome during episodes of ARI.

The level of knowledge of the various aspects of ARI was significantly associated with sociodemographic variables in some cases. More respondents younger than 30 had good knowledge of the symptoms and causes of ARI, whereas more of those older than 30 had good knowledge of the danger signs of severe ARI. The reason for this difference is not immediately apparent, although younger caregivers are more likely to have better education and as such more complete information from healthcare providers than older caregivers.

Although there were fewer male than female caregivers, a larger proportion of male caregivers displayed good or fair knowledge about the symptoms and causes of ARI. This may be due to men being employed outside the home more often than women and so could give them access to information more readily. This situation could lead to negative implications for the outcome of ARI in under-fives, as most of the caregivers were female. Although the majority of caregivers had secondary education, proportionately more of those with good knowledge of symptoms, causes and danger signs of severe ARI were educated to tertiary level. The association between educational status and knowledge level was not consistently statistically significant, although the larger proportion of those with tertiary education having good knowledge was expected. However, it should be noted that a larger proportion of respondents with primary education had good knowledge of the symptoms and causes of ARI compared with those with secondary education. The reason for this is not clear and was contrary to the expected trend. This may affect the management of ARI in this community.

The majority of the respondents were of a mid-level socioeconomic status, a reflection of the occupation status of the respondents (civil servants, petty traders or farmers). Knowledge of the various aspects of ARI was not significantly associated with socioeconomic status.

We identified a need for educating this community about homebased treatment of ARI, as the remedies used included both harmless and harmful substances. The use of an oily substance or kerosene could lead to complications such as lipoid pneumonitis, which, in turn, can prolong the duration of the infection and result in worse outcomes in a simple case. In addition to homemade remedies, caregivers also indicated that they bought drugs from the chemist during episodes of ARI. Topical applications were most commonly used in our study, whereas Kibuule and Kagoya^[25]^ reported oral herbal remedies (unknown components) frequently being used elsewhere. The use of oral herbal remedies was not reported in our study.

Tea with a 1:1 mixture of honey and lemon juice has been reported as a common remedy in home treatment of ARI in Asian countries.^[26]^ Other ingredients commonly mentioned include tamarind, ginger and eucalyptus. The use of natural products like these is more likely to be patient-friendly than using products such as those seen in the Edaiken community in our study. The choice of home remedies may be related to cultural and religious beliefs.

## Conclusion

The knowledge of caregivers regarding the symptoms, causes and danger signs of ARI was poor in this community. Knowledge level was found to be associated with sociodemographic variables in some cases. Respondents indicated that various remedies, often including the use of oily substances such as shea butter oil, palm kernel oil or kerosene, are used in the home-based treatment of ARI in under-fives. It is recommended that healthcare workers deliver regular education with regard to common childhood conditions and practical homebased remedies among the Edaiken community.
